# Inaccuracy in the Scientific Record and Open Postpublication Critique

**DOI:** 10.1177/17456916221141357

**Published:** 2023-02-06

**Authors:** Chris R. Brewin

**Affiliations:** Research Department of Clinical Educational & Health Psychology, University College London

**Keywords:** publication, citation, error, peer review

## Abstract

There is growing evidence that the published psychological literature is marred by multiple errors and inaccuracies and often fails to reflect the changing nature of the knowledge base. At least four types of error are common—citation error, methodological error, statistical error, and interpretation error. In the face of the apparent inevitability of these inaccuracies, core scientific values such as openness and transparency require that correction mechanisms are readily available. In this article, I reviewed standard mechanisms in psychology journals and found them to have limitations. The effects of more widely enabling open postpublication critique in the same journal in addition to conventional peer review are considered. This mechanism is well established in medicine and the life sciences but rare in psychology and may assist psychological science to correct itself.

Despite many impressive achievements, there are good reasons to fear that the scientific record in psychology contains a high number of errors. Multiple studies in other academic fields have attested to the high prevalence of mistakes in published articles, such as incorrect claims and statistical errors (Goldacre et al., 2019). The record is also constantly changing. How individual experiments should be interpreted alters with the arrival of exact and conceptual replications that test the robustness and generalizability of their effects (Patil et al., 2016; D. J. Stanley & Spence, 2014; Yarkoni, 2022). Applications and implications of data can be constrained by new findings as well. Yet there is evidence that citation patterns fail to reflect this changing state of affairs (Serra-Garcia & Gneezy, 2021; Tatsioni et al., 2007). The cost introduced by such errors is almost certainly considerable.

The aim of the current article is to categorize some of the errors frequently encountered in published psychology articles, summarize evidence showing such errors are likely to be common, consider their consequences, and discuss possible strategies to mitigate their effects. I argue that it could be advantageous to have a mechanism that allowed the psychological community to rapidly correct errors in published articles and initiate discussion about them online. Such mechanisms, part of a broader practice known as “postpublication peer review,” are becoming well established in medicine and the life sciences. Examples of postpublication peer review are readily available in a range of online forums and blogs concerned with making science more open and accountable. A more specific mechanism is postpublication critique, defined by Hardwicke et al. (2022, Supplementary Information K) as “any journal-based avenue for sharing peer-initiated critical discourse related to specific research articles previously published in the same journal.” With the occasional exception (Harms & Crede, 2020), such remedial strategies have not been discussed in mainstream psychology journals.

## Inaccuracy in the Scientific Record

The different forms of inaccuracy can be conveniently summarized under four main headings corresponding to the different stages of conducting a piece of research: summarizing past literature, carrying out the investigation, analyzing the results, and interpreting the findings. Inaccuracy that favors a particular perspective has been referred to as “spin” (Fletcher & Black, 2007), suggesting a conscious or unconscious wish to present results in the best possible light, but other errors may be simple mistakes.

### Citation error

This can take two main forms. “Biased citation” involves a mischaracterization of the relevant literature through citations that do not capture the current state of knowledge or do not direct the reader to reliable sources of information. A common form of biased citation, the selective reporting of studies or individual study findings, has also been referred to as “dissemination bias.” One way of demonstrating biased citation is by examining citations to articles that have been retracted. Several studies in biomedicine (Candal-Pedreira et al., 2020; Piller, 2021; Schneider et al., 2020) and psychology (Morís Fernández et al., 2019) have found that retraction does not prevent positive citations continuing to occur. Biased citation is also reflected in the expectation that landmark studies are mentioned less often following failures to replicate them, with the replication results being acknowledged and explicitly debated in subsequent articles. Evidence to date suggests that this process of correction does not typically happen in the psychological literature (Hardwicke et al., 2021; Schafmeister, 2021; Serra-Garcia & Gneezy, 2021; von Hippel, 2022).

The other form is “quotation inaccuracy,” which involves attributing specific views or findings to articles that they did not actually contain. This has been extensively studied in the medical literature. A meta-analytic summary suggested that in 100 cited references, readers could expect approximately 11% of quotations would contain major errors so severe that they were not at all in accordance with what the original authors claimed (Jergas & Baethge, 2015). Another recent review article considered the accuracy of quoted “facts” and suggested that about 10% involved major errors in which the referenced source either failed to substantiate, was unrelated to, or contradicted the assertion (Mogull, 2017). Similar systematic analyses of the psychology literature are rare, but examination of the way specific articles have been cited has revealed high levels of misquotation (Andrews & Brewin, 2017; Eagly, 2011; Harzing, 2002; Martella et al., 2021), including in the description of classic articles (Vicente, 2000). Another important form of quotation inaccuracy is the misstating or the drawing of incorrect inferences from psychological theories.

### Methodological error

This refers to faults in the way a study is planned or conducted or in the way this is reported. At the simplest level, errors have been documented in the recording of psychological data and their transcription (Rosenthal, 1978), an issue that can also affect the extraction of data for meta-analyses (Mathes et al., 2017). Common methodological problems include improper designs, invalid and unreliable measurement, confounding, inappropriate handling of missing data, lack of blinding or other biasing factors, and failures to follow a protocol. Each area of research is likely to be vulnerable to specific types of error. For example, common problems have been documented in family and marital research (O’Leary & Turkewitz, 1978) and in the measurement of the relation between confidence and accuracy in eyewitness identification (Wixted et al., 2015). Methods may also be described incorrectly in published articles, sometimes presenting a more favorable picture than is the case (Boutron & Ravaud, 2018).

A general criticism of much psychological research is that it is underpowered such that the small numbers of participants studied greatly restrict the opportunities to demonstrate the effect of interest even when it exists in the population. Reports have suggested that the average power to detect medium size effects in social psychology and personality research, or intelligence research, is around 50% (Fraley & Vazire, 2014; Nuijten et al., 2020). Examination of 200 meta-analyses published in *Psychological Bulletin* revealed that the median power across areas of research was about 36%, and only about 8% of studies had adequate power using Cohen’s 80% convention (T. D. Stanley et al., 2018).

### Statistical error

This involves mistakes in the correct application and reporting of statistical tests on the data collected. Systematic studies have suggested that almost one fifth of results based on null hypothesis significance testing (NHST) in the psychological literature are incorrectly reported and that around 15% of articles contain at least one statistical conclusion that is incorrect (Bakker & Wicherts, 2011). A subsequent study similarly reported that one in eight of all published psychology articles that used NHST contained a grossly inconsistent *p* value that may have affected the statistical conclusion (Nuijten et al., 2016). Furthermore, serious concerns have been raised about the practice (“p-hacking”) of making the decision about which part to publish after scrutinizing the data and reporting only that part of a data set that yields significant results (Simonsohn et al., 2014).

These issues help to account for the difficulty that is often found in reproducing published results from the raw data (Artner et al., 2021; Hardwicke et al., 2018; Maassen et al., 2020). Another general issue is the selective reporting of analyses that favor one particular interpretation of the data (Boutron & Ravaud, 2018). More specific problems have been identified in the reporting of regression-based mediation analyses in high-impact journals within the domain of personality and social psychology (Petrocelli et al., 2013) and in the testing of assumptions required for linear regression (Bullock & Green, 2021; Ernst & Albers, 2017). Examination of negative results reported in more than 14,000 articles from eight major psychology journals found evidence for false negative findings in almost half (Hartgerink et al., 2017).

### Interpretation error

This involves drawing inappropriate inferences about what the data show or how they can be applied to other issues and questions (e.g., real-world applicability). It includes exaggerating or relying on *p* values to make claims about the strength of effects found (Boutron & Ravaud, 2018). Particular attention has recently been paid to the problem of overgeneralization in psychological science (Yarkoni, 2022). The issue is that testing of verbally expressed theories and constructs relies on their prior operationalization to generate quantitative findings. The gap between these operationalizations and the constructs they are designed to measure requires that findings are described with a level of precision that is often lacking. Yarkoni (2022) noted that inferences about broad theories are often drawn using experimentation that is extremely limited in terms of the type of participants tested, the stimulus materials used, or the manipulations employed. One area in which concerns have frequently been expressed over unwarranted inferences is the generalization of findings from laboratory studies of memory to the examination of memory for real-world crimes in legal settings (Brewin, 2022a; DePrince et al., 2004; Goodman et al., 2019).

### Consequences of inaccuracy

There is now compelling evidence that errors in all parts of published psychology articles are not only common but also often serious. Some of these are more preventable than others. Whereas the sheer volume of published articles makes it hard to keep aware of and provide an unbiased citation of all relevant research, most quotation inaccuracy is avoidable by studying original sources and not relying on secondhand accounts of research. Likewise, the development of statistical knowledge has inevitably revealed flaws in earlier publications that adhered to different standards. These articles may nevertheless continue to be influential despite having serious weaknesses such as insufficient power or inappropriate claims to have “proved” the null hypothesis. In contrast, procedures can be put in place to minimize errors in data extraction, coding, or reporting.

These errors have the potential to influence the assumptions and design of new studies, whether they are replications, tests of similar hypotheses in new contexts, or further investigations of theory and mechanism. A misplaced reliance on the trustworthiness of earlier published findings might lead research in the wrong direction or misinform future studies in other ways (e.g., by affecting power calculations). Concerningly, investigators frequently report that these errors are not random but appear biased toward supporting the authors’ hypotheses (Bakker & Wicherts, 2011; Bishop, 2020; Hosseini et al., 2020; Rosenthal, 1978). This is consistent with observations that a high level of investment in certain hypotheses can lead to authors ignoring, criticizing, or suppressing nonsupportive data (Ferguson, 2015) and failing to update their views as new data emerge (Hardwicke et al., 2021; Serra-Garcia & Gneezy, 2021; Tatsioni et al., 2007).

The implication is that errors have the capacity to undermine or nullify self-correcting influences within the scientific process, resulting not only in a considerable waste of research dollars but also of the time involved in studying the literature, identifying new research questions, designing studies, and preparing grant applications. There is the further potential to mislead students and other groups who depend on psychological expertise, such as health-service personnel and lawyers (Brewin & Andrews, 2019; Brewin et al., 2019). Leaving errors uncorrected therefore involves professional, educational, reputational, financial, and scientific risk to the psychological community.

## Mitigating the Effects of Published Errors

In theory, authors, reviewers, and editors all bear responsibility for allowing incorrect accounts of research to enter the public domain. Making the content of traditional prepublication peer review (i.e., the reviewers’ and editor’s comments) accessible to all can do much to help identify the strengths and weaknesses of the scrutiny afforded to a particular article. Readers can identify what critical issues have already been raised, whether any errors have been detected, and the authors’ response and get a sense of the thoroughness and fairness of a journal’s peer-review practices. However, it is unrealistic to expect reviewers and editors to have the breadth of knowledge and time required to eliminate all errors. Likewise, the documented insensitivity of the literature to new findings underscores the practical difficulty of reviewers being able to keep up to date with a complex and rapidly expanding knowledge base. Retraction of an article can be difficult to discover (Schneider et al., 2020) despite the existence of dedicated websites such as Retraction Watch, although this may improve as databases such as Endnote and Web of Science begin to identify retracted articles. It is time to recognize the inevitability of errors, whether motivated or not, and consider how they can be corrected as quickly and effectively as possible. To be useful, the method of correction should be as undemanding of time and resources as possible (Vazire & Holcombe, 2022).

Some de facto correction mechanisms already exist. For example, systematic reviews and meta-analyses may overcome the limitations of individual studies by drawing on multiple sources of evidence to generate a more reliable picture of the effects obtained. Citing these rather than individual studies may reduce the incidence of citation bias. But reviews and meta-analyses themselves may be prone to error (Harris et al., 2019; Zhou et al., 2021). Likewise, smart citation indices, such as scite_, report more nuanced information about whether citing articles simply mention a study or actually provide supporting or contradictory information. But these approaches cannot provide the detailed critique and identification of errors that are necessary to inform readers. One solution is open peer commentary, which involves a set of reviews that is published simultaneously with a target article. This may identify errors but is a relatively inflexible system that can be used with only a small number of articles and does not permit uninvited comments.

An alternative approach involves some form of open postpublication peer review. This can be relatively informal, using personal social media or web annotation, for example, but generally makes use of formal channels constructed for the purpose. There are several platforms that host reviews on any scientific article. For example, PubPeer.com moderates reviews, which are required to consist of logic, facts, or publicly verifiable information. Comments, which may be anonymous, are not reviewed for scientific content, and readers are encouraged to evaluate them for themselves. Authors are notified of reviews and encouraged to respond. ScienceOpen.com similarly enables reviews of any published article to be posted, but reviewers are named. Hypothes.is enables the annotating of articles on the web, and PREreview provides for the structured open review of preprints. MyCites (Hosseini et al., 2020) is a proposed tool that would allow ORCID users to publicly mark and correct quotation inaccuracies in any publication, automatically generating notices to the journal, the cited authors, and the authors of the citing article.

For reviews and comments to have maximum impact and visibility, however, there is a strong case for them being hosted on a website owned by individual journals, where they are more likely to be found by readers of the original article. The most common form of this postpublication critique, at least among leading journals in psychiatry and psychology (Hardwicke et al., 2022), is for the same journal to publish unsolicited letters or commentaries in a subsequent issue. Within psychology, commentaries are generally the preferred approach. Although some leading journals (e.g., *Psychological Bulletin*) have a stated policy of not permitting or encouraging unsolicited commentaries on articles that have appeared in print, others, such as the *Journal of Psychopathology and Clinical Science* (formerly the *Journal of Abnormal Psychology*), acknowledge their role but require that they contain original data. The policy of the *Journal of Applied Psychology* clarifies that commentaries are judged against a high bar of making a substantive scientific contribution and that corrections of factual errors are resolved by publication of a one-page statement by the original authors that is then associated with the electronic version of the article (Kozlowski, 2011).

In *Psychological Science*, commentaries require new data or a reanalysis of existing data, but a more flexible format is available in the form of Letters to the Editor. These brief contributions are published online as supplementary information to the original article and are subject to an accelerated review process by the editorial team to determine whether they further scientific exchange (Bauer, 2021). Individuals may have only one letter published, and restrictions exist about the time frame within which letters are accepted and their length.

This brief survey illustrates that the scope for commentaries, the primary form of postpublication critique in psychology journals, is generally quite restricted. Even if allowed, they may be resisted by editors because these comments might sometimes bear on their own judgment and that of the earlier reviewers they invited to comment (Allison et al., 2016; Friedman et al., 2020; Goldacre et al., 2019). Consistent with this, there is some evidence of bias in decisions made by editors of biomedical journals (Scanff et al., 2021). Mechanisms that rely on the original authors submitting a correction or update depend on the authors’ objectivity and understanding of scientific procedures, which may not always be reliable (Goldacre et al., 2019; Vazire & Holcombe, 2022). A variety of other problems have been identified (Allison et al., 2016): Where to send expressions of concern is often unclear, such concerns are often overlooked, and some journals may charge authors to correct others’ mistakes. Thus, few, if any, of the mechanisms available to most psychology journals appear adequate to correct the large number and variety of errors, and in some cases they may impede publication of serious doubts about the validity and tenability of published results.

Open postpublication critique is rare in psychology compared with areas such as clinical medicine and biology/biochemistry (Hardwicke et al., 2022). The *British Medical Journal* and *PLOS ONE*, for example, supplement traditional prepublication peer review with a Responses tab associated with each online article that enables the free uploading of community comment. There are a number of issues that need to be thought through before deciding whether such an approach, used in conjunction with traditional peer review, would be an advantage to journals in psychology. It is likely that publications will have differing views about what is the most appropriate mechanism given their content and readership. For example, journals in mathematical psychology might value different commentators and different kinds of comment from general-theory journals or journals in clinical or educational psychology.

## Issues With Open Postpublication Critique

### Frequency

An initial question is how often postpublication critiques could be anticipated. At present, it appears that only around 5% of articles in leading psychology and psychiatry journals attract some form of critique (Hardwicke et al., 2022). This figure is likely to be a lower bound given the current restrictions on making postpublication comments. It suggests, however, that initial use of open online critique would be modest, although greater use could be anticipated with increased familiarity and with the advent of easily accessible response channels.

Given the pressure of requests for conventional peer review, how many researchers will have the time or inclination to use such a resource? It is likely that most will be motivated to comment only on articles that are especially influential or that address topics they are currently pursuing and that comments will be heavily clustered in the most active research areas. However, arguably these are the articles that would most benefit from being scrutinized for possible errors or for the additional insights that could be provided by individuals who were not involved in the prepublication peer review. This form of postpublication critique has the potential to open up to a wider audience discussions and contrasting views that at present take place, if they happen at all, on specialist community platforms.

### Gatekeeping

Perhaps the first decision is whether comments are permitted to be anonymous or must be by named individuals. If anonymous, there is a danger that postpublication critique might sometimes be used to continue existing disagreements or express opinions that are not specifically to do with the published article. If individuals are named, there could be an issue about commentators concealing their true identity or even masquerading as other people. Journals would also have to decide whether there should be any restrictions on individuals who can post comments (e.g., members of the academic community). A solution would be to accept comments from verifiable email addresses at academic institutions. However, although this would widen participation to include undergraduate and graduate students, it might exclude retired academics and qualified others without a current institutional affiliation.

ScienceOpen.com requires commentators on already published articles to have five records associated with their ORCID account to demonstrate that they are active professional researchers. Arguably, many errors could be identified by individuals without any of these qualifications, who might have additional insights to share. For example, the *British Medical Journal* and *The Lancet* sometimes involve as reviewers patients, carers of patients, or patient advocates to increase the relevance and patient-centeredness of their articles. This is an approach that could be considered especially by journals in applied psychology.

Some degree of moderation would be required to determine relevance; to exclude inappropriate content, such as ad hominem remarks, allegations of misconduct, and speculation about researcher actions and motive; and to ensure language was polite and neutral. Beyond that, criteria could vary considerably. Following the PubPeer.com model, comments could be confined to logic, facts, or publicly verifiable information. Alternatively, following the *British Medical Journal* model, comments might be permitted to include more broadly based opinions or evaluations. For example, commentators might pose questions about missing or unclear detail, suggest alternative analytic strategies, or raise valid issues of interpretation based on their own personal experience. Editorial boards would be well placed to specify the type of commentary that would be useful for individual journals.

Another issue concerns possible restrictions on content, number of submissions, or time elapsed since publication of the original article, which are commonly employed with published letters and commentaries. Given that errors may come to light only considerably later, that types of error are very varied, and that productive discussions may sometimes require several rounds of back-and-forth responses, the greatest flexibility would be achieved by the absence of any restrictions. One cost to this would be that checks would need to be made periodically to ensure that errors had not subsequently been found in regularly cited articles.

### Accessibility and citability

Various models are available that allow different levels of retrievability and citability of postpublication critique. At present, there is no standard mechanism for alerting readers of articles in psychology journals to the existence of subsequent commentaries or correspondence. In addition to such alerts for postpublication critiques, a tab for comments associated with each article would facilitate an immediate awareness of online responses.

At minimum, comments can be made freely available on journal-article webpages without being otherwise citable. At *Psychological Science*, Letters to the Editors are assigned Digital Object Identifiers (DOIs) but are not indexed (i.e., discoverable through PubMed, PsycInfo, etc.). At the *British Medical Journal*, submitted electronic comments have their own URL and are retrievable in a search of bmj.com. A selection of these comments is published as letters and indexed in PubMed. On ScienceOpen.com, reviews are published with a Creative Commons Attribution License CC-BY (4.0) license and also receive a DOI from CrossRef, similar to a formal research publication. This means that reviews are citable and able to be integrated with databases of individual research activity, such as ORCID or Publons.

### Costs

Some modest initial costs connected with the modification of journal websites would be inevitable. To this should be added costs attributable to hosting, archiving, and assigning digital identifiers to the additional reviews. However, online commentary is a low-cost solution compared with the publication of formal commentaries or letters. The workload of journal editors would increase somewhat with the need to monitor submitted comments, depending on how restrictive the journal requirements were. If necessary, an associate editor could be appointed to manage this aspect of the journal’s activities. Legal advice might occasionally be necessary.

### Validity

Systematic evaluations of the effectiveness of conventional peer review versus postpublication critiques are currently lacking. There seem to be a number of possible points of comparison. First, if the invited peer reviewers’ comments are published, subsequent postpublication comments might disagree with them or simply be inconsistent with them. Independent scrutiny could then assess the relative validity of the two sets of comments. Second, if the invited peer reviewers’ comments are not published, subsequent postpublication comments might imply that the decision to publish was unsound and that the article should be retracted (Knoepfler, 2015). It is an open question whether postpublication critique results in more valid decisions or more valid comments overall than are provided by conventional peer review.

A third possibility, and one that is strongly supported by the evidence reviewed in the section Inaccuracy in the Scientific Record, is that postpublication critique will address errors that were not detected by conventional peer review but that would not necessarily have affected the decision to publish. This is because the potential pool of postpublication reviewers will contain all the experts and other knowledgeable individuals who were not asked or who were unable to review the submission. The very breadth of the interests represented makes it highly plausible that they will identify some issues that the invited reviewer (and editor) panel did not. However, it is also the case that some of the postpublication contributions may be ill informed, incorrect, or based on reviewer bias. This should be addressed in future research.

### Cultural change

One major consequence is that authors would have to get used to their work being publicly appraised for possible errors and to receiving reviews that were more often negative than positive (Knoepfler, 2015). Systematic studies of providing authors with critical public feedback have documented how currently this is often resisted, sometimes vigorously (Goldacre et al., 2019). Yet such debate is arguably healthy for individual professional development and essential if psychology is to correct itself. Moreover, it is a change that has already been largely anticipated by the expectation that data and code will be made publicly available and by the increasing use of preprint servers.

In the life sciences, errors have been detected in important, controversial articles and reported almost immediately in outlets devoted to postpublication review. For example, Knoepfler (2015) described how in 2014 two articles on so-called STAP (stimulus-triggered acquisition of pluripotency) cells were published in *Nature* reporting a seemingly too-good-to-be-true method of cellular reprogramming. These articles were the focus of postpublication critiques on PubPeer and other sites, leading to the retraction of those articles and correction of the scientific record within a few months. Knoepfler commented that if the articles had been published 5 or 10 years ago, it would probably have taken several years for the record to be corrected, during which time valuable resources might have been squandered and trainee careers placed in jeopardy.

A recent study investigated whether corrected or retracted articles in several disciplines were associated with either subsequent negative citations as classified by scite_ or postpublication comments on PubPeer (Bordignon, 2020). Such articles were found to be associated with only an increased number of PubPeer comments, suggesting that postpublication review may have had a role to play in the later correction or withdrawal of the articles.

Knoepfler (2015) also noted how ongoing scientific debates between leading protagonists in biology and life sciences have been played out on PubPeer and on preprint servers, such as bioRxiv, with questions about articles being posed and answered almost in real time. The opportunities afforded for this kind of detailed methodological probing are rare in psychology. A recent example concerned the question of which articles should be included in a review of whether traumatic memories are fragmented and disorganized in individuals with posttraumatic stress disorder (McNally et al., 2022). An exchange of views about the admissibility of different studies eventually appeared in print 6 years after the initial articles were published and led to a reanalysis of the data based on the resulting insights (Brewin, 2022b). The existence of publicly accessible forums in which controversial issues were promptly debated by their protagonists could revolutionize the speed with which psychological science progresses.

The introduction of postpublication critique can be expected to be slow initially. If it is generally available for most journals, however, a culture change may be anticipated in which online comments come to assume greater importance and to be routinely monitored (Knoepfler, 2015). This could have potential benefits for all the people, including editors, reviewers, researchers, and undergraduate and graduate students, who are attempting to evaluate the strengths and weaknesses of specific key articles. More generally, psychologists are likely to become more aware of the difficulty of avoiding all error, accepting this in their own work and that of others, and adapting their behavior accordingly.

## Conclusion

There is increasing evidence that standard safeguards such as prepublication peer review are inadequate to prevent a substantial level of error in scientific publication. Realistically, we as psychologists can no longer expect publications, whether our own or those authored by others, to be entirely error free. Given that some level of error appears to be predictable, core scientific values such as openness, transparency, and commitment to accuracy require that we consider how to issue corrections as swiftly and effectively as possible. Relatedly, the Committee on Publication Ethics (2022), whose members include major publishers such as the American Psychological Association, Elsevier, Sage, Springer Nature, Taylor & Francis, and Wiley, stipulates that “journals must allow debate post publication either on their site, through letters to the editor, or on an external moderated site, such as PubPeer. They must have mechanisms for correcting, revising or retracting articles after publication.” The spirit, and usually the letter, of this stipulation appears to have been largely ignored.

Scientific disciplines vary greatly in their approach to these issues (Hardwicke et al., 2022; Walker & da Silva, 2015), and some version of open postpublication review as employed in medicine and the life sciences may be a realistic option for psychology. A wide variety of solutions are possible, such as online Letters to the Editor or open discussion on same-journal or specialist websites. Such practices have been put forward as one indicator by which scientific communities can be evaluated on their success at achieving self-correction (Vazire & Holcombe, 2022).

Open same-journal postpublication critique is therefore, at least in theory, an attractive mechanism for rapidly identifying errors, raising methodological issues, and drawing attention to subsequent relevant research, including positive and negative replications. Some design decisions may have wide applicability, for example, making comments submitted online easy to upload, retrieve, and cite, and eliminating restrictions on the time, length, or number of comments. Authors should be able to easily respond where appropriate and either accept or rebut comments as well as spontaneously correct and update their own work. Other decisions concerning content or gatekeeping are more likely to depend on the aims and readership of specific journals.

Although it is possible that some forms of postpublication critique bring with them disadvantages, such changes could potentially facilitate and raise the profile of debate; expose students, teachers, and researchers to alternative perspectives; enable busy authors, reviewers, and editors to more accurately assess the status of cited research; and reduce the waste associated with flawed science. Such a resource is likely to be especially useful in areas of high current interest and in applied areas in which many studies are difficult or impossible to replicate.

## Transparency

*Action Editor:* Klaus Fiedler

*Editor:* Klaus Fiedler
